# Supplementary material for: Cortical Thickness Changes in Migraine Patients Treated with Anti-Calcitonin Gene-Related Peptide Monoclonal Antibodies: A Prospective Age- and Sex-Matched Controlled Study
Source: Biomedicines. 2025 May 9;13(5):1150. doi: 10.3390/biomedicines13051150 (PMC12108776; doi:10.3390/biomedicines13051150)
Supplement: Supplementary file 1 [file biomedicines-13-01150-s001.zip › biomedicines-3594171-supplementary.pdf]

**Supplementary materials**

Supplementary figure S1. Pipeline of cortical thickness measurement using the ATROSCAN platform and representative brain renderings and quantitative outputs illustrating regional cortical thickness in a migraine patient.

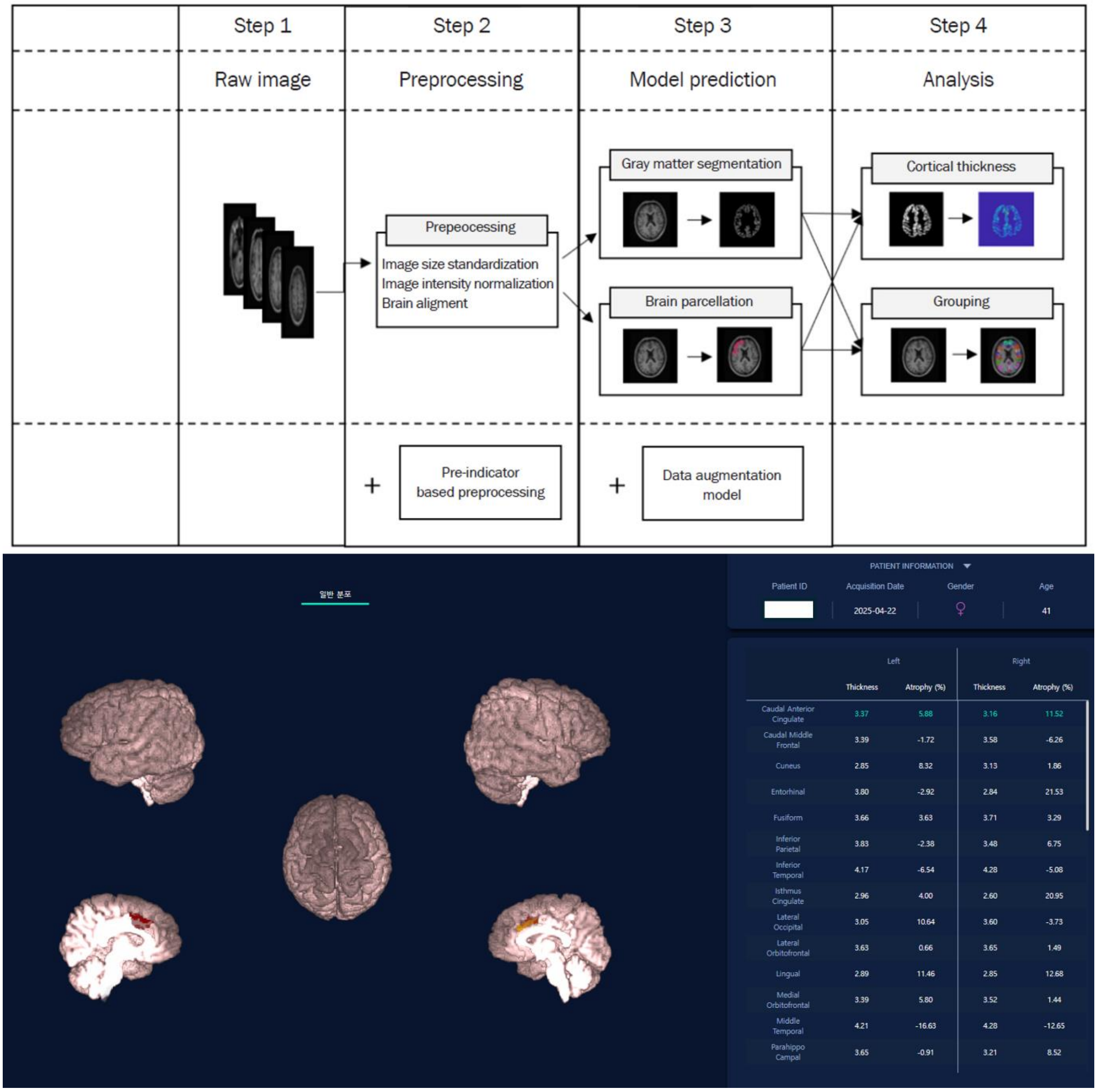


Supplementary Table S1. Cortical thickness changes across 46 brain regions in the anti-CGRP treatment group and the oral treatment group

| | Oral treatment group (n=15) | | | Anti-CGRP treatment (n=15) | | | p* |
|---|---|---|---|---|---|---|---|
| | Baseline | After 3 months treatment | Change in cortical thickness | Baseline | After 3 months treatment | Change in cortical thickness | |
| Left caudal anterior cingulate cortex | 3.02 (2.95-3.16) | 3 (2.79-3.21) | -0.1 (-0.22-0.08) | 3.27 (2.9-3.37) | 3.16 (2.89-3.49) | -0.07 (-0.17-0.29) | 0.519 |
| Left caudal middle frontal cortex | 3.42 (3.38-3.54) | 3.45 (3.32-3.57) | 0 (-0.12-0.18) | 3.6 (3.47-3.69) | 3.62 (3.46-3.83) | 0.01 (-0.12-0.22) | 0.927 |
| Left cuneus cortex | 2.35 (2.26-2.44) | 2.36 (2.2-2.41) | -0.04 (-0.08-0.07) | 2.43 (2.29-2.57) | 2.48 (2.29-2.58) | 0.04 (-0.07-0.15) | 0.198 |
| Left entorhinal cortex | 3.31 (3.19-3.52) | 3.32 (3.14-3.48) | -0.03 (-0.08-0.17) | 3.46 (3.25-3.67) | 3.34 (3.24-3.63) | 0.07 (-0.23-0.19) | 0.645 |
| Left fusiform cortex | 3.37 (3.32-3.53) | 3.38 (3.28-3.47) | 0.04 (-0.19-0.28) | 3.42 (3.2-3.6) | 3.41 (3.29-3.58) | 0.02 (-0.13-0.24) | >0.999 |
| Left inferior parietal cortex | 3.27 (3.17-3.44) | 3.27 (3.09-3.35) | -0.02 (-0.2-0.14) | 3.34 (3.11-3.45) | 3.34 (3.2-3.5) | 0.05 (-0.04-0.19) | 0.157 |
| Left inferior temporal cortex | 3.85 (3.74-3.97) | 3.9 (3.72-4) | -0.01 (-0.15-0.18) | 3.89 (3.71-4.04) | 3.97 (3.87-4.09) | 0.07 (-0.01-0.17) | 0.329 |
| Left isthmus cingulate cortex | 2.43 (2.26-2.56) | 2.39 (2.31-2.55) | -0.01 (-0.11-0.05) | 2.42 (2.3-2.51) | 2.42 (2.28-2.44) | -0.03 (-0.13-0.06) | 0.751 |
| Left lateral occipital cortex | 2.93 (2.86-3.01) | 2.97 (2.88-3.07) | 0.06 (-0.07-0.14) | 3.07 (2.94-3.11) | 2.99 (2.97-3.11) | 0 (-0.12-0.16) | 0.645 |
| Left lateral orbitofrontal cortex | 3.47 (3.4-3.59) | 3.47 (3.34-3.71) | 0 (-0.22-0.21) | 3.54 (3.39-3.77) | 3.59 (3.4-3.76) | 0.05 (-0.15-0.11) | 0.846 |
| Left lingual cortex | 2.5 (2.33-2.63) | 2.43 (2.36-2.64) | 0.03 (-0.07-0.1) | 2.39 (2.33-2.62) | 2.53 (2.44-2.62) | 0.01 (-0.01-0.21) | 0.506 |
| Left medial orbitofrontal cortex | 3.03 (2.95-3.18) | 3.22 (3.01-3.29) | 0.09 (0.03-0.19) | 3.13 (2.73-3.36) | 3.07 (2.71-3.13) | -0.04 (-0.2-0.28) | 0.395 |
| Left middle temporal cortex | 3.87 (3.64-3.91) | 3.85 (3.72-4.06) | 0.11 (-0.09-0.16) | 3.92 (3.76-4.06) | 3.94 (3.78-4.04) | 0.06 (-0.07-0.17) | 0.878 |
| Left parahippocampal cortex | 3.125 (2.91-3.35) | 3.09 (2.93-3.27) | -0.04 (-0.21-0.29) | 3.09 (2.86-3.31) | 3.19 (2.92-3.32) | -0.01 (-0.14-0.25) | 0.675 |
| Left paracentral cortex | 2.58 (2.38-2.83) | 2.59 (2.42-2.74) | -0.03 (-0.12-0.16) | 2.71 (2.43-2.8) | 2.64 (2.43-2.82) | -0.05 (-0.12-0.13) | 0.943 |
| Left pericalcarine cortex | 2.165 (2.05-2.25) | 2.09 (2.05-2.17) | -0.01 (-0.14-0.11) | 2.23 (2.16-2.28) | 2.18 (2.11-2.27) | -0.02 (-0.1-0.08) | 0.959 |
| Left posterior cingulate cortex | 2.65 (2.58-2.78) | 2.81 (2.52-3.12) | 0.09 (-0.26-0.33) | 2.76 (2.49-3.02) | 2.75 (2.57-3) | -0.02 (-0.26-0.3) | 0.862 |
| Left precuneus cortex | 2.64 (2.53-2.73) | 2.64 (2.52-2.73) | -0.03 (-0.13-0.08) | 2.79 (2.6-2.98) | 2.78 (2.6-2.93) | 0.03 (-0.12-0.3) | 0.467 |
| Left rostral anterior cingulate cortex | 3.15 (2.75-3.4) | 2.98 (2.77-3.14) | -0.19 (-0.49-0.24) | 3.13 (2.81-3.53) | 3.27 (2.82-3.71) | 0 (-0.07-0.18) | 0.351 |
| Left rostral middle frontal cortex | 3.81 (3.71-3.93) | 3.77 (3.64-3.86) | -0.03 (-0.15-0.05) | 3.83 (3.75-4.01) | 3.86 (3.61-3.96) | -0.12 (-0.21-0.12) | 0.675 |
| Left supramarginal cortex | 3.11 (3.03-3.23) | 3.21 (3.06-3.3) | 0.07 (-0.09-0.27) | 3.14 (2.98-3.32) | 3.23 (3.14-3.35) | 0.07 (-0.09-0.25) | 0.943 |
| Left transverse temporal cortex | 2.54 (2.35-2.8) | 2.65 (2.38-2.86) | 0.1 (-0.14-0.21) | 2.62 (2.56-2.83) | 2.69 (2.59-2.79) | 0.1 (-0.1-0.19) | 0.767 |
| Left insula cortex | 3.48 (3.17-3.57) | 3.35 (3.15-3.52) | -0.06 (-0.36-0.26) | 3.51 (3.27-3.7) | 3.58 (3.29-3.7) | 0.02 (-0.18-0.17) | 0.690 |
| Right caudal anterior cingulate cortex | 3.15 (3.01-3.5) | 3.23 (3.09-3.37) | 0.04 (-0.22-0.15) | 3.24 (3.08-3.31) | 3.14 (2.92-3.44) | -0.12 (-0.29-0.18) | 0.560 |

| | | | | | | | |
|---|---|---|---|---|---|---|---|
| Right caudal middle frontal cortex | 3.56 (3.34-3.6) | 3.52 (3.28-3.94) | 0.08 (-0.12-0.19) | 3.57 (3.32-3.71) | 3.65 (3.54-3.83) | 0.02 (-0.13-0.24) | 0.814 |
| Right cuneus cortex | 2.34 (2.24-2.47) | 2.4 (2.27-2.44) | 0.01 (-0.07-0.1) | 2.47 (2.22-2.57) | 2.39 (2.27-2.66) | 0.01 (-0.1-0.21) | 0.645 |
| Right entorhinal cortex | 3.28 (3.11-3.43) | 3.2 (3.02-3.41) | 0.05 (-0.26-0.14) | 3.38 (2.91-3.5) | 3.38 (3.16-3.57) | 0.15 (-0.22-0.25) | 0.395 |
| Right fusiform cortex | 3.33 (3.2-3.41) | 3.3 (3.21-3.52) | 0.01 (-0.2-0.16) | 3.33 (3.17-3.45) | 3.35 (3.2-3.49) | -0.01 (-0.15-0.32) | 0.645 |
| Right inferior parietal cortex | 3.39 (3.29-3.6) | 3.44 (3.32-3.57) | 0.02 (-0.28-0.14) | 3.42 (3.24-3.67) | 3.44 (3.34-3.63) | 0.06 (-0.17-0.17) | 0.546 |
| Right inferior temporal cortex | 4.1 (3.91-4.16) | 4.02 (3.89-4.12) | -0.03 (-0.21-0.12) | 4.14 (3.88-4.22) | 4.04 (3.87-4.24) | 0.03 (-0.14-0.17) | 0.645 |
| Right isthmus cingulate cortex | 2.44 (2.24-2.52) | 2.4 (2.28-2.59) | 0.05 (-0.21-0.13) | 2.44 (2.31-2.51) | 2.45 (2.35-2.57) | 0.1 (-0.14-0.13) | 0.573 |
| Right lateral occipital cortex | 3.04 (2.94-3.25) | 3.08 (2.97-3.17) | -0.06 (-0.18-0.1) | 3.13 (2.93-3.25) | 3.09 (2.99-3.44) | 0.04 (-0.13-0.17) | 0.290 |
| Right lateral orbitofrontal cortex | 3.4 (3.27-3.5) | 3.43 (3.32-3.5) | 0.03 (-0.12-0.18) | 3.43 (3.26-3.47) | 3.38 (3.23-3.59) | 0.05 (-0.1-0.19) | 0.782 |
| Right lingual cortex | 2.49 (2.46-2.57) | 2.54 (2.38-2.59) | -0.02 (-0.12-0.07) | 2.43 (2.32-2.61) | 2.54 (2.46-2.64) | 0.1 (-0.1-0.19) | 0.183 |
| Right medial orbitofrontal cortex | 3.04 (2.98-3.16) | 3.06 (2.93-3.13) | 0.01 (-0.17-0.06) | 3.07 (2.86-3.18) | 3.02 (2.91-3.3) | 0.01 (-0.18-0.18) | 0.660 |
| Right middle temporal cortex | 3.71 (3.44-3.92) | 3.66 (3.45-3.78) | 0.03 (-0.22-0.12) | 3.82 (3.52-3.9) | 3.92 (3.57-4) | 0.14 (-0.07-0.26) | 0.139 |
| Right parahippocampal cortex | 2.94 (2.77-3.17) | 2.85 (2.77-3.02) | -0.05 (-0.26-0.16) | 3 (2.83-3.11) | 2.94 (2.8-3.12) | -0.01 (-0.23-0.24) | 0.506 |
| Right paracentral cortex | 2.45 (2.36-2.69) | 2.48 (2.38-2.56) | -0.03 (-0.28-0.16) | 2.51 (2.36-2.84) | 2.48 (2.4-2.56) | 0 (-0.15-0.07) | 0.894 |
| Right pericalcarine cortex | 2.11 (2.01-2.32) | 2.08 (2.05-2.12) | -0.01 (-0.22-0.05) | 2.18 (2.02-2.29) | 2.11 (2.06-2.23) | -0.07 (-0.15-0.19) | >0.999 |
| Right posterior cingulate cortex | 2.46 (2.42-2.75) | 2.59 (2.38-2.67) | -0.02 (-0.14-0.19) | 2.64 (2.48-2.92) | 2.57 (2.4-2.78) | 0.02 (-0.31-0.25) | >0.999 |
| Right precuneus cortex | 2.63 (2.52-2.82) | 2.7 (2.51-2.82) | 0 (-0.24-0.28) | 2.8 (2.52-2.87) | 2.76 (2.65-2.93) | 0.1 (-0.14-0.25) | 0.532 |
| Right rostral anterior cingulate cortex | 3.06 (2.79-3.27) | 2.98 (2.83-3.15) | 0.04 (-0.29-0.15) | 3.25 (3.06-3.4) | 3.21 (2.95-3.49) | 0.02 (-0.19-0.16) | 0.602 |
| Right rostral middle frontal cortex | 3.84 (3.56-4.08) | 3.89 (3.69-4.08) | 0.04 (-0.19-0.15) | 3.88 (3.6-4.04) | 3.95 (3.79-4.06) | 0.03 (-0.11-0.21) | 0.430 |
| Right supramarginal cortex | 3.19 (3.02-3.26) | 3.12 (2.99-3.22) | -0.02 (-0.2-0.05) | 3.19 (2.97-3.35) | 3.13 (3.04-3.26) | 0.03 (-0.21-0.1) | 0.493 |
| Right transverse temporal cortex | 2.65 (2.35-2.83) | 2.51 (2.38-2.68) | 0 (-0.31-0.13) | 2.57 (2.49-2.73) | 2.7 (2.52-2.88) | 0.06 (-0.08-0.1) | 0.418 |
| Right insula cortex | 3.31 (3.1-3.54) | 3.18 (2.95-3.35) | 0 (-0.23-0.05) | 3.4 (3.11-3.67) | 3.43 (3.29-3.57) | -0.11 (-0.26-0.29) | 0.862 |

Values are presented as median (interquartile range).

*The p-values indicate the between-group difference in cortical thickness change (anti-CGRP vs. oral treatment), assessed using the Mann–Whitney U test.

Supplementary Table S2. Cortical thickness changes across 46 brain regions in the non-responders and responders to anti-CGRP mAbs

| | Non-responders (n=6) | | | Responders (n=9) | | | p* |
|---|---|---|---|---|---|---|---|
| | Baseline | After 3 months treatment | Change in cortical thickness | Baseline | After 3 months treatment | Change in cortical thickness | |
| Left caudal anterior cingulate cortex | 3.3 (2.87-3.4) | 3.59 (3.07-3.72) | 0.27 (0.1-0.39) | 3.19 (2.86-3.38) | 3.14 (2.74-3.33) | -0.16 (-0.34-0.05) | 0.312 |
| Left caudal middle frontal cortex | 3.52 (3.37-3.6) | 3.76 (3.58-3.86) | 0.23 (-0.02-0.48) | 3.66 (3.51-3.7) | 3.57 (3.4-3.79) | -0.03 (-0.16-0.1) | 0.323 |
| Left cuneus cortex | 2.37 (2.09-2.51) | 2.31 (2.21-2.78) | 0.06 (-0.08-0.28) | 2.45 (2.33-2.63) | 2.5 (2.42-2.55) | 0.03 (-0.15-0.15) | 0.509 |
| Left entorhinal cortex | 3.4 (3.19-3.61) | 3.34 (3.28-3.64) | 0.07 (-0.1-0.26) | 3.46 (3.29-3.68) | 3.34 (3.07-3.69) | 0.07 (-0.33-0.14) | 0.372 |
| Left fusiform cortex | 3.33 (3.18-3.52) | 3.44 (3.33-3.59) | 0.08 (-0.04-0.27) | 3.57 (3.18-3.67) | 3.41 (3.21-3.61) | -0.07 (-0.3-0.25) | 0.388 |
| Left inferior parietal cortex | 3.3 (3.17-3.48) | 3.51 (3.2-3.66) | 0.13 (0.05-0.21) | 3.35 (3.1-3.46) | 3.28 (3.22-3.4) | -0.01 (-0.07-0.14) | 0.081 |
| Left inferior temporal cortex | 3.85 (3.67-3.95) | 3.96 (3.86-4.12) | 0.09 (0.05-0.36) | 3.98 (3.75-4.05) | 3.99 (3.79-4.12) | 0.07 (-0.16-0.17) | 0.438 |
| Left isthmus cingulate cortex | 2.48 (2.38-2.55) | 2.41 (2.26-2.46) | -0.06 (-0.17--0.03) | 2.41 (2.28-2.47) | 2.42 (2.27-2.49) | 0.06 (-0.1-0.12) | 0.107 |
| Left lateral occipital cortex | 3.03 (2.94-3.11) | 3 (2.97-3.15) | 0.04 (-0.05-0.09) | 3.08 (2.85-3.13) | 2.99 (2.93-3.14) | -0.06 (-0.13-0.2) | 0.798 |
| Left lateral orbitofrontal cortex | 3.53 (3.3-3.75) | 3.7 (3.39-3.83) | 0.1 (0.02-0.28) | 3.54 (3.4-3.82) | 3.5 (3.33-3.75) | -0.11 (-0.18-0.07) | 0.150 |
| Left lingual cortex | 2.47 (2.34-2.62) | 2.57 (2.42-2.69) | 0.05 (-0.04-0.24) | 2.39 (2.3-2.7) | 2.53 (2.44-2.6) | 0.01 (-0.16-0.26) | 0.889 |
| Left medial orbitofrontal cortex | 3.03 (2.7-3.24) | 3.08 (2.94-3.23) | 0.08 (-0.13-0.36) | 3.18 (2.73-3.38) | 2.98 (2.66-3.22) | -0.12 (-0.24-0.21) | 0.328 |
| Left middle temporal cortex | 3.87 (3.56-4.01) | 4.04 (3.67-4.19) | 0.09 (-0.03-0.35) | 3.92 (3.81-4.07) | 3.91 (3.78-4) | 0.02 (-0.12-0.13) | 0.342 |
| Left parahippocampal cortex | 2.9 (2.82-3.15) | 3.1 (2.9-3.34) | 0.11 (-0.08-0.36) | 3.26 (2.93-3.44) | 3.2 (2.95-3.4) | -0.06 (-0.15-0.18) | 0.313 |
| Left paracentral cortex | 2.71 (2.51-2.8) | 2.73 (2.42-3.16) | 0.03 (-0.07-0.41) | 2.71 (2.42-2.85) | 2.63 (2.41-2.78) | -0.08 (-0.23-0.05) | 0.136 |
| Left pericalcarine cortex | 2.24 (2.11-2.28) | 2.2 (2.1-2.34) | 0.02 (-0.1-0.12) | 2.22 (2.18-2.29) | 2.18 (2.11-2.27) | -0.02 (-0.15-0.05) | 0.371 |
| Left posterior cingulate cortex | 2.83 (2.55-3.13) | 3.02 (2.61-3.43) | 0.15 (-0.02-0.4) | 2.76 (2.48-3.04) | 2.72 (2.46-2.91) | -0.06 (-0.33-0.19) | 0.082 |
| Left precuneus cortex | 2.77 (2.47-2.91) | 2.84 (2.67-3.22) | 0.22 (-0.08-0.34) | 2.79 (2.62-2.99) | 2.68 (2.58-2.92) | 0 (-0.21-0.08) | 0.145 |
| Left rostral anterior cingulate cortex | 3.27 (3.01-3.57) | 3.68 (3.1-3.76) | 0.08 (-0.05-0.54) | 3.13 (2.81-3.56) | 2.98 (2.65-3.41) | 0 (-0.4-0.13) | 0.272 |
| Left rostral middle frontal cortex | 3.88 (3.61-4.03) | 3.97 (3.79-4.1) | 0.06 (-0.07-0.28) | 3.83 (3.76-4.04) | 3.73 (3.6-3.89) | -0.17 (-0.22--0.1) | 0.007 |
| Left supramarginal cortex | 3.04 (2.96-3.19) | 3.24 (3.14-3.43) | 0.19 (0.03-0.39) | 3.25 (2.98-3.43) | 3.16 (3.09-3.34) | -0.09 (-0.12-0.15) | 0.062 |
| Left transverse temporal cortex | 2.73 (2.57-2.84) | 2.74 (2.47-2.89) | 0.06 (-0.23-0.17) | 2.6 (2.49-2.79) | 2.67 (2.6-2.73) | 0.1 (-0.07-0.2) | 0.626 |
| Left insula cortex | 3.38 (3.18-3.61) | 3.69 (3.31-3.83) | 0.12 (-0.1-0.5) | 3.57 (3.28-3.74) | 3.5 (3.28-3.64) | -0.12 (-0.26-0.09) | 0.145 |
| Right caudal anterior cingulate cortex | 3.16 (2.9-3.4) | 3.45 (3.2-3.62) | 0.13 (-0.01-0.46) | 3.26 (3.1-3.38) | 2.99 (2.87-3.18) | -0.17 (-0.34--0.11) | 0.026 |

| | | | | | | | |
|---|---|---|---|---|---|---|---|
| Right caudal middle frontal cortex | 3.58 (3.34-3.68) | 3.78 (3.66-3.9) | 0.24 (0.04-0.44) | 3.52 (3.3-3.98) | 3.56 (3.24-3.67) | -0.06 (-0.27-0.09) | 0.139 |
| Right cuneus cortex | 2.34 (2.16-2.47) | 2.36 (2.26-2.78) | 0.21 (-0.07-0.31) | 2.54 (2.28-2.71) | 2.39 (2.31-2.59) | -0.01 (-0.3-0.15) | 0.113 |
| Right entorhinal cortex | 3.44 (3.21-3.59) | 3.54 (3.31-3.63) | 0.08 (-0.12-0.33) | 3.23 (2.9-3.47) | 3.31 (3.11-3.5) | 0.15 (-0.24-0.26) | 0.667 |
| Right fusiform cortex | 3.36 (3.26-3.53) | 3.37 (3.25-3.64) | -0.04 (-0.14-0.29) | 3.32 (3.09-3.4) | 3.27 (3.14-3.47) | 0.03 (-0.22-0.35) | 0.864 |
| Right inferior parietal cortex | 3.59 (3.34-3.68) | 3.55 (3.36-3.88) | 0.1 (-0.05-0.2) | 3.4 (3.23-3.63) | 3.38 (3.29-3.55) | -0.12 (-0.21-0.19) | 0.477 |
| Right inferior temporal cortex | 4.15 (3.98-4.25) | 4.2 (4.03-4.33) | 0.03 (-0.04-0.28) | 4.07 (3.74-4.22) | 3.93 (3.81-4.06) | -0.07 (-0.28-0.16) | 0.404 |
| Right isthmus cingulate cortex | 2.42 (2.27-2.47) | 2.49 (2.3-2.63) | 0.11 (0.02-0.24) | 2.44 (2.31-2.69) | 2.44 (2.36-2.6) | 0.04 (-0.22-0.13) | 0.258 |
| Right lateral occipital cortex | 3.15 (3.04-3.21) | 3.14 (3.1-3.46) | 0.13 (-0.05-0.28) | 3.13 (2.9-3.37) | 3.02 (2.83-3.3) | -0.04 (-0.15-0.14) | 0.171 |
| Right lateral orbitofrontal cortex | 3.41 (3.28-3.54) | 3.55 (3.27-3.64) | 0.08 (-0.09-0.2) | 3.44 (3.14-3.47) | 3.37 (3.17-3.48) | -0.03 (-0.1-0.19) | 0.604 |
| Right lingual cortex | 2.57 (2.34-2.61) | 2.47 (2.4-2.83) | 0.01 (-0.11-0.23) | 2.42 (2.31-2.68) | 2.56 (2.44-2.62) | 0.1 (-0.06-0.18) | 0.976 |
| Right medial orbitofrontal cortex | 3.18 (2.91-3.29) | 3.19 (3-3.63) | 0.07 (-0.24-0.52) | 3.03 (2.8-3.11) | 2.94 (2.7-3.18) | -0.05 (-0.16-0.12) | 0.607 |
| Right middle temporal cortex | 3.77 (3.35-3.96) | 3.93 (3.43-4.26) | 0.19 (-0.08-0.4) | 3.85 (3.61-3.89) | 3.92 (3.64-3.97) | 0.03 (-0.08-0.23) | 0.456 |
| Right parahippocampal cortex | 3.1 (2.97-3.12) | 3.03 (2.85-3.25) | -0.05 (-0.17-0.18) | 2.84 (2.74-3.1) | 2.87 (2.72-3.08) | 0 (-0.34-0.36) | 0.840 |
| Right paracentral cortex | 2.41 (2.21-2.85) | 2.52 (2.34-2.86) | 0.05 (-0.01-0.16) | 2.55 (2.42-2.8) | 2.47 (2.41-2.56) | -0.11 (-0.2-0.04) | 0.053 |
| Right pericalcarine cortex | 2.09 (1.5-2.23) | 2.11 (2.08-2.16) | 0.05 (-0.14-0.67) | 2.18 (2.13-2.3) | 2.13 (2.06-2.27) | -0.12 (-0.17-0.09) | 0.286 |
| Right posterior cingulate cortex | 2.6 (2.11-3.05) | 2.63 (2.32-2.91) | 0.09 (-0.16-0.26) | 2.77 (2.51-2.86) | 2.57 (2.48-2.78) | -0.22 (-0.34-0.2) | 0.607 |
| Right precuneus cortex | 2.65 (2.41-2.89) | 2.9 (2.59-3.13) | 0.23 (0.07-0.34) | 2.8 (2.55-2.93) | 2.71 (2.65-2.84) | -0.13 (-0.17-0.18) | 0.053 |
| Right rostral anterior cingulate cortex | 3.35 (3.01-3.53) | 3.54 (3.11-3.77) | 0.14 (0.01-0.3) | 3.23 (2.98-3.34) | 3.16 (2.88-3.28) | -0.04 (-0.24-0.07) | 0.150 |
| Right rostral middle frontal cortex | 3.91 (3.75-4.14) | 3.96 (3.86-4.23) | 0.04 (-0.16-0.28) | 3.73 (3.57-3.99) | 3.92 (3.64-4.05) | 0.03 (-0.11-0.32) | 0.797 |
| Right supramarginal cortex | 3.18 (2.89-3.27) | 3.23 (3.01-3.48) | 0.09 (-0.06-0.35) | 3.24 (3.02-3.39) | 3.09 (3.03-3.21) | -0.09 (-0.25-0.07) | 0.145 |
| Right transverse temporal cortex | 2.59 (2.46-2.76) | 2.65 (2.48-2.82) | 0.07 (-0.02-0.12) | 2.56 (2.49-2.79) | 2.7 (2.54-2.89) | 0.06 (-0.1-0.25) | 0.978 |
| Right insula cortex | 3.27 (3.1-3.53) | 3.39 (3.24-3.58) | 0.09 (-0.08-0.3) | 3.63 (3.1-3.81) | 3.46 (3.14-3.59) | -0.23 (-0.31-0.16) | 0.113 |

Values are presented as median (interquartile range).

*The p-values indicate the between-group difference in cortical thickness change (non-responders vs. responders), assessed using the Mann–Whitney U test.
